# Descriptive Analysis on the Impacts of Universal Zero-Markup Drug Policy on a Chinese Urban Tertiary Hospital

**DOI:** 10.1371/journal.pone.0162795

**Published:** 2016-09-14

**Authors:** Wei Tian, Jiangfan Yuan, Dong Yang, Lanjing Zhang

**Affiliations:** 1 Department of Spine Surgery, Jishuitan Hospital, Beijing, China; 2 Office of Patient-Physician Relationship, Jishuitan Hospital, Beijing, China; 3 Office of Performance Management, Jishuitan Hospital, Beijing, China; 4 Department of Pathology, University Medical Center of Princeton, Plainsboro, NJ, United States of America; 5 Rutgers Cancer Institute of New Jersey, New Brunswick, NJ, United States of America; 6 Department of Pathology, Robert Wood Johnson Medical School, Rutgers University, New Brunswick, NJ, United States of America; 7 Department of Chemical Biology, Ernest Mario School of Pharmacy, Rutgers University, Piscataway, NJ, United States of America; Old Dominion University, UNITED STATES

## Abstract

**Background:**

Universal Zero-Markup Drug Policy (UZMDP) mandates no price mark-ups on any drug dispensed by a healthcare institution, and covers the medicines not included in the China’s National Essential Medicine System. Five tertiary hospitals in Beijing, China implemented UZMDP in 2012. Its impacts on these hospitals are unknown. We described the effects of UZMDP on a participating hospital, Jishuitan Hospital, Beijing, China (JST).

**Methods:**

This retrospective longitudinal study examined the hospital-level data of JST and city-level data of tertiary hospitals of Beijing, China (BJT) 2009–2015. Rank-sum tests and join-point regression analyses were used to assess absolute changes and differences in trends, respectively.

**Results:**

In absolute terms, after the UZDMP implementation, there were increased annual patient-visits and decreased ratios of medicine-to-healthcare-charges (RMOH) in JST outpatient and inpatient services; however, in outpatient service, physician work-days decreased and physician-workload and inflation-adjusted per-visit healthcare charges increased, while the inpatient physician work-days increased and inpatient mortality-rate reduced. Interestingly, the decreasing trend in inpatient mortality-rate was neutralized after UZDMP implementation. Compared with BJT and under influence of UZDMP, JST outpatient and inpatient services both had increasing trends in annual patient-visits (annual percentage changes[APC] = 8.1% and 6.5%, respectively) and decreasing trends in RMOH (APC = -4.3% and -5.4%, respectively), while JST outpatient services had increasing trend in inflation-adjusted per-visit healthcare charges (APC = 3.4%) and JST inpatient service had decreasing trend in inflation-adjusted per-visit medicine-charges (APC = -5.2%).

**Conclusion:**

Implementation of UZMDP seems to increase annual patient-visits, reduce RMOH and have different impacts on outpatient and inpatient services in a Chinese urban tertiary hospital.

## Introduction

China initiated a huge and complex health reform including the National Essential Medicine System (NEMS, also called national essential medicine policy) in 2009.[1] As detailed in one of the recent works, the NEMS covered 307 generic drugs including 205 Western medicines and 102 traditional Chinese medicines.[2] The NEMS also mandated that government-owned healthcare institutions must sell essential medicines with no mark-ups (hence also termed “zero mark-up drug policy” [ZMDP]).[2] Studies have shown that implementation of the NEMS reduced retail medicine-prices and availability of some medicines. [2–6] Not surprisingly, the data from Jiangxi and Shaanxi provinces and nation-level data of all provinces also show that the NEMS, particularly the ZMDP for essential medicines, led to significant reduction in per-visit medicine charges in rural areas. [3, 4, 7] Additional effects are physician prescription-pattern changes and differences in medicine prices between urban and rural healthcare institutions.[1, 2, 8] However, the increase or decrease of the medicine or healthcare charges was largely dependently on the hospital levels as Wang et al. showed.[9] Specifically, they found that both outpatient and inpatient charges were reduced in township health centers, but increased in the hospitals of county levels or above. It would be interesting to explore the effects of ZMDP on the annual inpatient- and outpatient-visit volumes in urban tertiary hospitals.

In 2012, as part of the Separation of Healthcare and Medicine Policy, Beijing City of China started a pilot program of implementing a universal ZMDP in 5 tertiary hospitals, including Chaoyang Hospital, Friendship Hospital, Jishuitan Hospital (JST), Tiantan Hospital and Tongren Hospital. Per this universal ZMDP, any medicines dispensed at the participating hospitals should have no price mark-ups and the financial loss would be in part recovered by the governmental compensations and increases in healthcare charges. For example, the old 4-tier outpatient healthcare registration and healthcare fees of RMB 5 (USD $0.76), RMB 7 (USD $1.07), RMB 9 (USD $1.37) and RMB 14 (USD $2.13) (the latter 3 for expert consultants’ services) were replaced by the new 4-tier outpatient health service fees according to the caregiver’s academic rank. The new 4-tier fee schedule for each visit included RMB 42 (USD $6.4), RMB 60 (USD $9.14), RMB 80 (USD $12.19) and RMB 100 (USD $15.23), significantly higher than the older schedule. Clearly, the universal ZMDP covers the medicines excluded by the NEMS and would have a broader impact on the local healthcare systems and patients than the NEMS. However, its effects on the participating hospitals are not clear. Therefore, we aimed to describe the changes in the hospital’s annual patient volumes, per-visit medicine charges, per-visit healthcare charges, ratio of the medicine over healthcare charges (RMOH), inpatient mortality and gross profit/loss after the implementation of universal ZMDP in JST in December 2012, and compared the aforementioned variables of JST with those of the tertiary hospitals of Beijing City (BJT) when available.

Increased outpatient and/or inpatient service volumes have been observed in some rural healthcare institutions after the implementation of ZMDP. [3, 7, 10] Given the well-known physician shortage issue in China (1.7 practicing physicians per 1,000 people in 2013, compared with the international average of 3.3) [11], it is possible that ZMDP will exaggerate the physician shortage and subsequently lead to increased physician-workload. However, no physician-workload differences were found before and after implementation of NEMS in township and county hospitals and/or health centers in 2009–2010, contrary to the expected increase in physician workload.[12] Therefore, our secondary aim was to describe the effects of the universal ZMDP on JST physician workload. Considering the association between increased physician workload and decreased healthcare quality and patient survivals, [13–19] we also planned to assess the changes of inpatient mortality, an important healthcare-quality metric, after the implementation of the universal ZMDP.

## Material and Methods

In this retrospective longitudinal descriptive study, we extracted the hospital-level data of the outpatient and inpatient services each year between 2009–2015 from the health information system of JST at Xinjiekou campus (excluding the Huilongguan campus), and the city-level data of all tertiary hospitals in Beijing City (BJT) each year between 2009–2015 from the annual *Briefings on the Health Statistics of Beijing City*.[20] The city-level data included the 5 tertiary hospitals where the ZMDP were implemented. The collected data included annual outpatient patient-visit volumes, annual inpatient patient-visit volumes, average healthcare charge per visit, average medicine charge per visit, RMOH, average per-physician outpatient encounter number per day, average per-physician inpatient patient-bed number per day, inpatient mortality rate, physician number, hospital bed-occupancy rate, and percentages of the total financial loss (gross loss) over total expenses. The average annual outpatient patient-visit volumes and inpatient discharge volumes of BJT were calculated by dividing the outpatient patient volume and inpatient discharges volumes of all BJT with the number of BJT in a given year. The RMOH was calculated by dividing average medicine charge per visit/discharge with average healthcare charge per visit/discharge.

The physician work-days in outpatient services and that in inpatient services were calculated using Eqs 1 and 2, respectively. In the equations, *wd*_*o*_ denotes outpatient work days, *v* annual outpatient patient-visit volume, *n* physician number, $\bar{p}$ average per-physician outpatient encounter-number per day, *wd*_*i*_ inpatient work-days, *h* hospital bed number, *o* occupancy rate of the hospital beds and $\bar{b}$ average per-physician patient-bed number per day. Since there is only one leap year (2012) in 2009–2015, for better consistency, we considered 365 days in each year, and decided to ignore the nominal one-day difference in the calculation. We also calculated the inflation-adjusted medicine and healthcare charges using the respective annual consumer product index (CPI) published by the National Bureau of Statistics of China[21].

$$wd_{o}=\frac{v}{\bar{p}\cdot n}$$ (1)

$$wd_{i}=\frac{h\cdot o\cdot 365}{\bar{b}\cdot n}$$ (2)

STATA IC version 11 (Stata Corp, College Station, TX, USA) was used for the statistical analyses as described before.[22] Wilcoxon rank-sum tests were used to compare the data of all involved variables before and after the universal ZDMP implementation. We also used the Joinpoint Regression Program (Version 4.2.0—April 2015; Statistical Methodology and Applications Branch, Surveillance Research Program, National Cancer Institute, Bethesda, MD, USA) to calculate and compare the annual percentage changes (APC) and average APC (AAPC). A meaningful join-point was considered being identified when there was a significant APC change according to the modeling (*P*<0.05). A parallel pair-wise comparison and the maximum of 1 join-point were used in the modeling, without any supervision. The detailed modeling of the join-point regression was described before [23]. A *P* value <0.05 was considered statistically significant. All reported *P* values were 2-sided.

## Results

We were able to extract the aforementioned outpatient and outpatient service information of JST 2009–2015 and BJT 2009–2015 including the 5 pilot tertiary hospitals with implementation of the ZMDP. Approximately 830 of the 1137 medicines (73%) carried by JST were not included in the NEMS, but covered by the universal ZMDP from December 2012 to December 2015 with nominal variations among the years. Our rank-sum tests showed that, in absolute terms (Table 1), the outpatient healthcare charges of JST hospital were higher after the implementation of the universal ZMDP in 2012 than before (23.9% increase, median RMB 379.14 [USD $57.94] versus RMB 469.74 [USD $71.78], *P* = 0.0339), while the inflation-adjusted per-visit outpatient medicine charges remained similar. Similarly, the inpatient healthcare charges remained similar after the implementation of universal ZMDP in December 2012, while the inflation-adjusted per-discharge inpatient medicine charges were significantly decreased after the implementation of the universal ZMDP (33.3% reduction, median RMB 6413.79 [USD $980.12] versus RMB 4280.78 [USD $654.16], *P* = 0.0339). Therefore, it is not surprising that the RMOH in the JST outpatient and inpatient services were both significantly lower after 2012 (*P* = 0.0339). Similar findings were revealed using Student *t* test (S1 Table).

**Table 1:** Comparison of the effects associated with the drug-price zero-markup policy implemented in Dec 2012 at the Jishuitan hospital at Xinjiekou campus, Beijing, China (Wilcoxon Rank-sum test).

	2009–2012			2013–2015			
	25 percentile	median	75 percentile	25 percentile	median	75 percentile	*P* value (Mann-Whitney *U* test)
***Outpatient***							
**Volume**	1019291	1123859	1224251	1527099	1802014	1896189	0.0339
Healthcare charge (RMB)*	353.895	379.14	385.59	407.29	469.74	484.21	0.0339
Medicine charge (RMB)*	226.7325	242.72	247.9975	209.19	213.15	224.64	0.0771
**RMOH**	0.63475	0.641	0.648	0.454	0.464	0.514	0.0339
**Physician workload**	6.7625	6.97	7.1925	9.67	10.73	10.94	0.0339
**Physician work day**	254	257	261	249	249	251	0.0339
***Inpatient***							
**Volume**	27768.75	31053	37460.25	42622	49576	50880	0.0339
Healthcare charge (RMB)*	25335.38	27167.66	29903.21	26095.5	26885.99	29153.44	>0.99
Medicine charge (RMB)*	6131.93	6413.79	6695.917	3912.11	4280.78	4830.67	0.0339
**RMOH**	0.2195	0.2305	0.2535	0.134	0.159	0.185	0.0339
**Physician workload**	2.155	2.405	2.4975	2.38	2.6	2.63	0.1573
**Mortality**	0.0059	0.0077	0.0087	0.0028	0.0037	0.0038	0.0339
**Physician work day**	241	247	248	269	304	337	0.0339
***Gross loss over total expenses***	-13.1317%	-11.8713%	-9.7449%	-17.3094%	-14.06%	-13.5621%	0.077

Note:

*Adjusted by the respective annual consumer product indices in China with that in 2009 as 100%

RMB, Reminbi; Physician workloads are average patient-encounter number per day for outpatient service and average patient-bed number per day for inpatient service, respectively.

**Fig 1:**
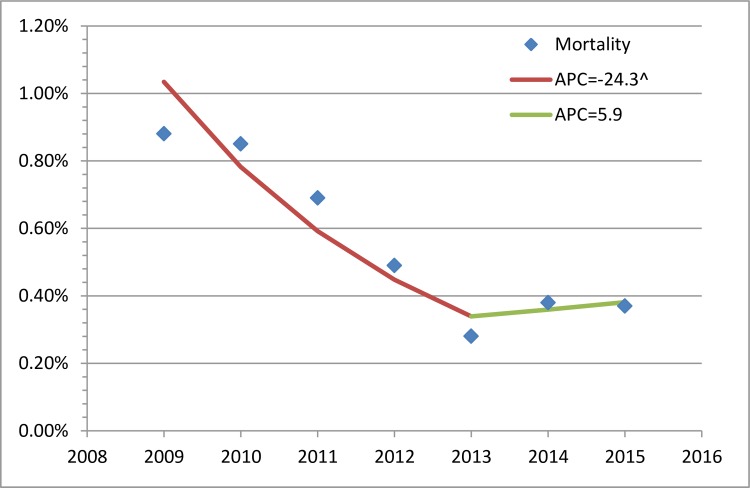
Trends in the outpatient and inpatient services of Jishuitan Hospital at Xinjiekou campus, Beijing, China (JST) and tertiary hospitals of Beijing, China (BJT). ^: a significant trend identified (*P*<0.05); APC: Annual percentage change; RMOH: Ratio medicine over health charges. Physician workloads: average annual patient-visit number per day for outpatient service and average patient-bed number per day for inpatient service, respectively.

Table 1 also shows that the annual patient-visit volumes of the JST outpatient and inpatient services were both significantly increased after 2012 (*P* = 0.0339 for both), and the inpatient mortality rates reduced (*P* = 0.0339). However, the JST outpatient physician workload (per-physician outpatient patient encounter per day) was significantly increased after 2012 (*P* = 0.0339) while the JST inpatient physician workload (per-physician inpatient patient-bed per day) remained similar (*P* = 0.1573). We then calculated the average physician work-days, which decreased for the outpatient services but increased for the inpatient services after 2012 (*P* = 0.0339 for both, Table 1). We found that the inpatient mortality rate of JST was decreasing each year till 2013 (APC 24.3%, *P* = 0.029, Table 2 and Fig 1). Of note, despite the neutralization of the decreasing trend, the JST inpatient mortality remained lower after 2012 than 2012 and before (*P* = 0.02339, Table 1).

**Table 2:** The trends in the inpatient mortality of Jishuitan Hospital at Xinjiekou campus, Beijing, China.

Segment		Lower Endpoint	Upper Endpoint	APC	Lower CI	Upper CI	*P*-Value
**1-JP model**	Slope 1	**2009**	**2013**	**-24.3^**	-38.6	-6.7	0.029
	Slope 2	2013	2015	5.9	-45.4	105.5	0.75
**No-JP model**	Full Range	2009	2015	**-16.7^**	-25.5	-6.8	0.009

Note: APC, annual percentage change; CI, 95% confidence interval; JP, Join point

^ and bold fonts indicate a *P* value<0.05.

We continued to observe gross losses in absolute terms (data not shown) after the implementation of the universal ZMDP. Due to the rising total revenues and expenses, the percentages of financial loss (gross loss) over total expenses seemed more meaningful than the absolute numbers for assessing the potential changes linked to the universal ZMDP implementation. No significant differences were found after implementation of the universal ZMDP using either a rank-sum or *Student* t test (Table 1 and S1 Table).

**Fig 2:**
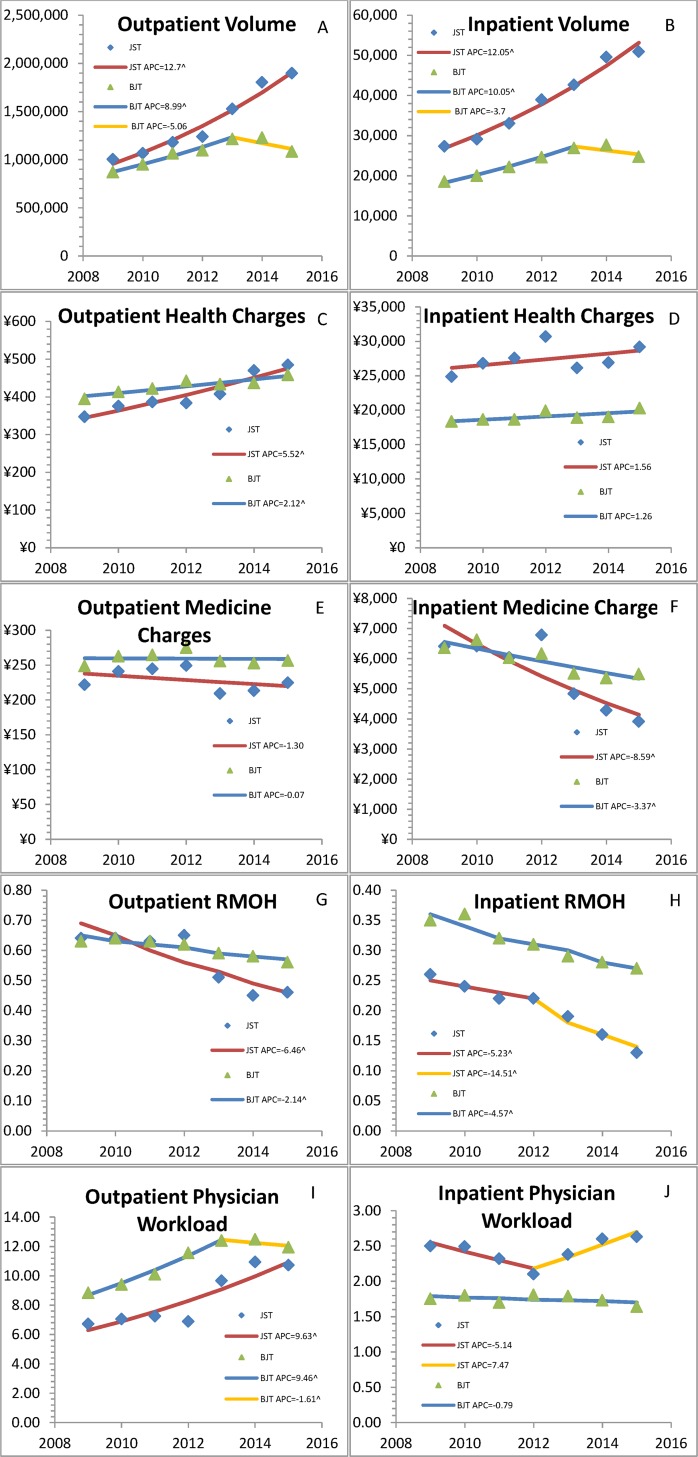
Trends in the inpatient mortality of Jishuitan Hospital at Xinjiekou campus, Beijing, China. Modeled Segment-1: a significant trend identified (annual percentage change[APC] = -24.31%, *P* = 0.029); Modeled Segment-2: no significant trends identified (*P* = 0.75).

**Fig 3:**
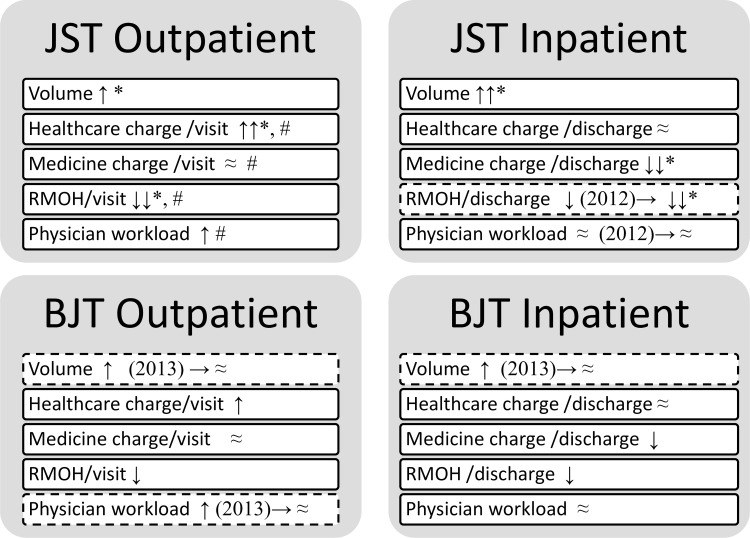
Summary of the differences in the trends of outpatient and inpatient services in Jishuitan hospital and the tertiary hospitals in Beijing, China. The years indicate the time segments with significant changes (*P*<0.05) revealed using the join-point regression analysis. The dash-lined box indicates significant differences in the trends; JST: Jishuitan Hospital at Xinjiekou campus, Beijing, China; BJT: The tertiary hospitals in Beijing, China; ≈: No significant trends identified (*P*>0.05); → a join-point identified (join-point year in parenthesis); ↑: significantly increased annual percentage changes (*P*<0.05); ↓: significantly decreased annual percentage changes (*P*<0.05); * significant difference in trends between Jishuitan hospital and the tertiary hospitals in Beijing; # significant difference in trends between outpatient and inpatient services of Jishuitan hospital.

According to the *Briefings on the health statistics of Beijing City*,[20] there were 50, 51, 51, 72, 79, 88 and 108 tertiary hospitals of Beijing City in 2009, 2010, 2011, 2012, 2013, 2014 and 2015, respectively. The joinpoint models were employed to estimate the potential differences in trends (DIT) in the aforementioned variables and physician-workloads of the outpatient or inpatient services between JST and BJT (Tables 3 and 4, and Figs 2 and 3). Compared with BJT and perhaps under influence of universal ZDMP, the JST outpatient and inpatient services both had increasing trends in annual patient-visit volumes (annual percentage changes [APC] = 8.1% and 6.5%, respectively, *P*<0.001 for both) and decreasing trends in RMOH (APC = -4.3%, *P* = 0.005 and -5.4%, *P*<0.001, respectively). Compared with BJT, the JST outpatient services had an increasing trend in inflation-adjusted per-visit healthcare charges (APC = 3.4%, *P*<0.001) and no DIT in inflation-adjusted per-visit medicine charges or physician workload (*P* = 0.40 and *P* = 0.134, respectively), while the JST inpatient service had a decreasing trend in inflation-adjusted per-visit medicine-charges (APC = -5.2%, *P* = 0.015) and no DIT in inflation-adjusted per-visit healthcare charges or physician workload (*P* = 0.831 and *P* = 0.295, respectively).

**Table 3:** The outpatient effects associated of the zero-markup drug policy implemented in Dec 2012 at the Jishuitan Hospital at Xinjiekou campus, Beijing, China in comparison with the Beijing tertiary hospitals.

JST						BJT						JST versus BJT			
Join-point	Segment range	APC	Lower CI	Upper CI	*P*-Value	Join-point	Segment range	APC	Lower CI	Upper CI	*P*-Value	APC	Lower CI	Upper CI	P-Value
**Volume**															
**0**	2009–2015	12.2^	9.4	15.1	<0.001	1	2009–2013	9.0^	3.2	15.1	0.021	**8.1**^	**4.2**	**12.0**	**<0.001**
							2013–2015	-5.1	-20.1	12.8	0.325				
Healthcare charge per visit/discharge*															
**0**	2009–2015	5.5^	3.4	7.6	0.001	0	2009–2015	2.1^	1.1	3.1	0.003	**3.4**^	**1.6**	**5.2**	**<0.001**
Medicine charge per visit/discharge*															
**0**	2009–2015	-1.3	-4.6	2.1	0.363	0	2009–2015	-0.1	-1.8	1.7	0.928	-1.2	-4.1	1.6	0.400
**RMOH**															
**0**	2009–2015	-6.5^	-10.2	-2.5	0.009	0	2009–2015	-2.1^	-3	-1.3	0.002	**-4.3**^	**-7.3**	**-1.3**	** 0.005**
**Physician workload**															
**0**	2009–2015	9.6^	4.2	15.3	0.005	1	2009–2013	9.4^	3.9	15.3	0.017	4.0	-1.2	9.3	0.134
							2013–2015	-1.6	-16.5	16	0.712				

Note: JST: Jishuitan Hospital; BJT: Beijing Tertiary Hospitals; APC, annual percentage change; AAPC, average annual percentage change; CI, 95% confidence interval; JP, Join point; RMOH, ratio of the medicine over healthcare charges; Physician workloads, average annual patient-visit number per day for outpatient service and average patient-bed number per day for inpatient service, respectively

* Adjusted by the respective annual consumer product indices in China with that in 2009 as 100%

^ indicates a *P* value<0.05

$ 1 indicates 1-join-point is identified (*P*<0.05).

**Table 4:** The inpatient effects associated of the zero-markup drug policy implemented in Dec 2012 at the Jishuitan Hospital at Xinjiekou campus, Beijing, China in comparison with the Beijing tertiary hospitals.

JST						BJT						JST versus BJT			
Join-point $	Segment range	APC	Lower CI	Upper CI	*P*-Value	Join-point$	Segment range	APC	Lower CI	Upper CI	*P*-Value	APC	Lower CI	Upper CI	*P*-Value
**Volume**															
**0**	2009–2015	12.1^	10.1	14.1	<0.001	1	2009–2013	10.6^	8.6	12.6	0.002	**6.5**^	**4.6**	**8.3**	**<0.001**
							2013–2015	-3.7	-9	2	0.105				
Healthcare charge per visit/discharge*															
**0**	2009–2015	1.6	-1.7	4.9	0.28	0	2009–2015	1.3	-0.1	2.6	0.063	0.3	-2.4	3.0	0.831
Medicine charge per visit/discharge*															
**0**	2009–2015	-8.6^	-13.6	-3.2	0.01	0	2009–2015	-3.4^	-5.2	-1.5	0.006	**-5.2**^	**-9.4**	**-1.0**	**0.015**
**RMOH**															
**1**	2009–2012	-5.2^	-7.4	-3.1	0.01	0	2009–2015	-4.6^	-5.7	-3.4	<0.001	**-5.4**^	**-6.5**	**-4.3**	**<0.001**
	2012–2015	-14.5^	-16.4	-12.5	0.001										
**Physician workload**															
**1**	2009–2012	-5.1	-13.6	4.2	0.136	0	2009–2015	-0.8	-2.4	0.9	0.272	1.8	-1.5	5.0	0.295
	2012–2015	7.5	-2.1	18	0.08										

Note: JST: Jishuitan Hospital; BJT: Beijing Tertiary Hospitals; APC, annual percentage change; AAPC, average annual percentage change; CI, 95% confidence interval; JP, Join point; RMOH, ratio of the medicine over healthcare charges; Physician workloads, average annual patient-visit number per day for outpatient service and average patient-bed number per day for inpatient service, respectively

* Adjusted by the respective annual consumer product indices in China with that in 2009 as 100%

^ indicates a *P* value<0.05

$ 1 indicates 1-join-point is identified (*P*<0.05).

Strikingly, compared with the JST inpatient services, the JST outpatient services had significant DIT in physician workload and in all examined variables except annual patient-visit volume (Table 5 and Fig 1). Specifically, there were higher APCs in inflation-adjusted per-visit healthcare charges (APC = 4.0, *P* = 0.01), inflation-adjusted per-visit medicine charges (APC = 7.3, *P* = 0.002), RMOH (APC = 3.6, *P* = 0.02) and physician workload (APC = 8.7, *P* = 0.001).

**Table 5:** Comparison of the outpatient and inpatient service changes associated of the zero-markup policy implemented in Dec 2012 at the Jishuitan Hospital at Xinjiekou campus, Beijing, China.

	Outpatient versus inpatient			
	AAPC	Lower CI	Upper CI	*P*-Value
**Volume**				
	0.1	-2.5	2.8	0.93
**Healthcare charge per visit/discharge**				
	**4.0^**	1.0	7.0	**0.01**
**Medicine charge per visit/discharge**				
	**7.3^**	2.6	12.0	**0.002**
**RMOH**				
	3.6^	0.6	6.6	** 0.020**
**Physician workload**				
	**8.7^**	3.5	13.9	**0.001**

Note: APC, annual percentage change; AAPC, average annual percentage change; CI, 95% confidence interval; JP, Join point; RMOH, ratio of the medicine over healthcare charges; Physician workloads, average annual patient-visit number per day for outpatient service and average patient-bed number per day for inpatient service, respectively

^ and bold fonts indicate a *P* value<0.05.

## Discussion

We here show that, in a Chinese urban tertiary hospital, JST, the implementation of the universal ZMDP in 2012 after establishment of the NEMS in China was associated with increased annual patient-visit volumes and decreased RMOH in both outpatient and inpatient services, but no changes in the percentages of the gross loss over the total expenses. It also linked to different consequences in the JST outpatient and inpatient services and a possible shift of physician workforces (workdays) from the outpatient service to the inpatient service. Compared with BJT, there were also an increasing trend in the annual patient-visit volumes and a decreasing trend in RMOH of the JST outpatient and inpatient services after the implementation of the universal ZMDP in 2012.

This is one of the first longitudinal descriptive studies on the 3-year effects of the universal ZMDP on an urban tertiary hospital after implementation of the NEMS in 2009. Our work appears somewhat valuable because most of the prior studies were cross-sectional, focused on the effects of the NEMS and its ZMDP on essential medicines, and concerned about the rural areas of China. [3, 7, 10] Of note, one 2-year longitudinal study showed that implementation of the ZMDP for essential medicines was associated with similar reductions in medicine charges and similar physician prescribing behavioral changes in the urban and rural primary care institutions of Hubei Province, China.[2] Two cross-sectional studies also investigated urban areas of China. One found decreased access to the physical facilities, increased outpatient visits and increased inpatient admissions in urban area after implementation of the NEMS (2008 versus 2011).[22] The other showed decreased medicine expenses per prescription (2007 versus 2010) in urban areas, and different changes in medicine expenses (2007 versus 2010) between the urban and rural areas of China.[1] Moreover, most of the prior works investigated the implementation of the NEMS and its ZMDP on essential medicines.[1–8, 12, 24] Little is known regarding the impacts caused by or associated with the new universal ZMDP, while the potential impacts may be boarder and more significant than the NEMS and its ZMDP due to the much wider coverage of the medicines (100% versus 27% in JST). Therefore, our work may advance the understanding on the consequences of universal ZMDP, and provide useful data for future modifications of the universal ZMDP in addition to the NEMS and its ZMDP on essential medicines. Furthermore, a unique advantage of the longitudinal study is its ability to identify DIT and its impact on the trends, which, to our best knowledge, have not yet been analyzed.

Some of our findings are noteworthy and may help shape future policy-making on drug-prices in China. First, we provided a line of early evidence that the universal ZMDP is associated with increased annual patient-visits of the outpatient and inpatient services. Our findings are consistent with the increased inpatient patient-visits as reported before,[7, 22] and raise the concerns over potential over-use of the inpatient services and how to accommodate the increased demands on inpatient services. In the meantime, our findings are inconsistent with the unchanged outpatient patient-visits shown by one report and estimated a 2% reduction of outpatient visits shown by another.[7, 22] More data are needed on the changes of outpatient patient-visits. Despite the increased annual outpatient and inpatient services, the percentages of the gross loss over total expenses remained similar after the implementation of the universal ZMDP. The finding suggests a smaller profit loss per patient encounter. However, only the short-term (3-year) impact of the universal ZMDP was examined in this study, and the long-term impact is still unclear. Moreover, the expenses changes of a public hospital are complex and multifactorial. A detailed revenue-and-expenses analysis is probably beyond the scope of this study, but is a very interesting subject for future studies.

Second, the universal ZMDP appears to lead to different changes in the inflation-adjusted per-discharge medicine charges in outpatient and inpatient services. The finding is in contrast to the decreases in inflation-adjusted per-visit/discharge medicine charges of both outpatient and inpatient services in rural areas. [3, 7] Many factors may have contributed to the difference. One possible cause is that the universal ZMDP studied here covers all medicines dispensed by a healthcare institution, which is much more than the 307 essential medicines included in the NEMS. Another possible cause is that the various differences between the urban tertiary hospitals and the rural hospitals, town health centers and clinics, including the differences in patient socioeconomic status, culture norms, perception on drug prices, healthcare provider knowledge base and training, healthcare facility types and physician prescription patterns/habits.

Third, the percentage changes (-6% for 2013–2015 versus 2009–2012) of per-visit outpatient medicine charges after the implementation of the universal ZMDP seems to be fairly smaller than the allowable 15% markup cap. It is likely because that several factors contribute to the medicine charges per visit, including the number of medicine prescribed, the dosages (both strength and length of the medicine use) of the medicine prescribed, the drug-price markup, and patient/family compliance in purchasing medicines as instructed. Although the drug prices have been reduced under the universal ZMDP other factors such as the quantity of medicine purchased may contribute to the less than desired reduction in the per-visit outpatient medicine charges after the implementation of the universal ZMDP. Of course, it is also possible that the original drug-price markup in the outpatient services was lower than that in the inpatient services in additional to the aforementioned factors. Hence, the reduction became smaller than that in inpatient services (-30% for 2013–2015 versus 2009–2012). More studies are needed to validate and further elucidate of the findings of this descriptive analysis.

Finally, the implementation of the universal ZMDP in JST seems to link to an increase in physician workload of outpatient service in absolute terms and compared with that of inpatient service. However, compared with BJT, this increase in outpatient physician workload was no longer significant. The findings indicate such an increase is unlikely associated with the universal ZMDP implementation, but possibly with the overall changes of the BJT. Consistent with the few changes of physician workload, the inpatient mortality also seemed not to be severed by the implementation of the universal ZMDP. Future works may consider other healthcare quality metrics such as 30-day readmission rate and length of stay.[13, 16, 25–28]

**Fig 4:**
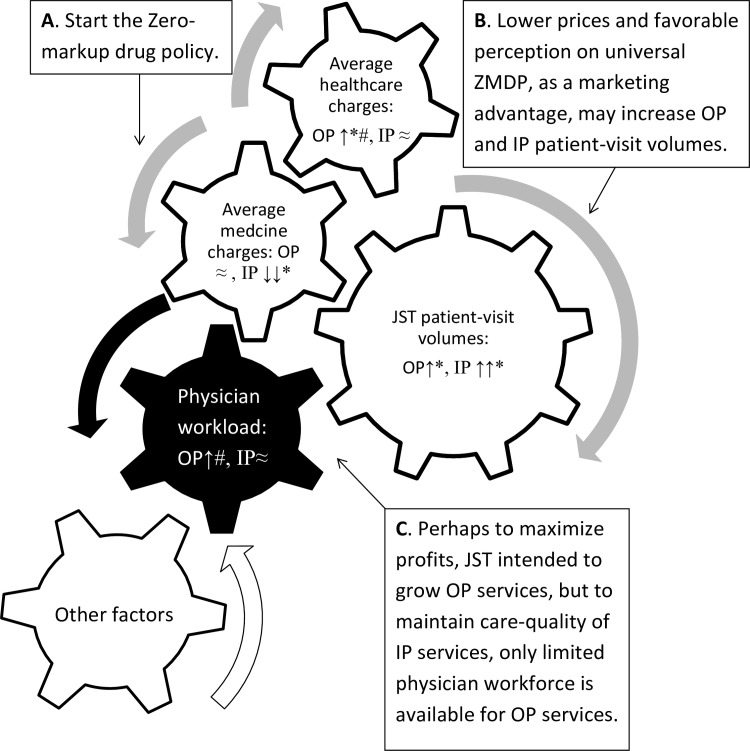
Theoretical framework. JST: Jishuitan Hospital at Xinjiekou campus, Beijing, China; OP: Outpatient; IP: Inpatient; ≈: No significant trends identified; ↑: significantly increased annual percentage changes (increasing trend); ↓: significantly decreased annual percentage changes (decreasing trend); * significant difference in trends between Jishuitan hospital and the tertiary hospitals in Beijing; # significant difference in trends between outpatient and inpatient services of Jishuitan hospital.

We here provide the following hypothesis to explain how universal ZMPD influences the moving parts of the healthcare market (Fig 4). **A**. The implementation of the universal ZMDP links to a decrease of per-discharge medicine charges in inpatient services, and decreased RMOH in outpatient and inpatient services. **B.** The relatively lower or decreasing medicine charges (prices in the market) and favorable perceptions on the universal ZMDP, as a marketing advantage, help increase the annual patient-visit volumes in outpatient and inpatient services. **C.** Perhaps to maximize profits, JST intended to grow outpatient services. But to maintain healthcare-quality of inpatient services, likely due to the concerns over potential malpractice lawsuits and the complexity of inpatient cases, inpatient services consume an increasingly bigger portion of physician worktime (more workdays) and outpatient services are allocated with an increasingly smaller portion of physician worktime. Therefore, compared to inpatient service, the physician workload of JST outpatient service increased, although neither of them had a significant DIT compared with the BJT counterparts. Finally, other factors may also contribute to the dynamic market changes associated with the implementation of the universal ZMDP.

Several potential limitations of this study should be considered. First, this is a single-institutional experience, despite also being a 7-year retrospective longitudinal study. The comparison with BJT is likely to increase the applicability and validity of our findings. Future studies of more urban hospitals with the universal ZMDP implementation are needed. Ideally, a set of fixed tertiary hospitals in Beijing should be compared with the 5 pilot hospitals with implementation of ZMDP. However, due to the lack of data granularity and technical constraints, it is almost impossible to exclude the 5 pilot hospitals from the rest of the tertiary hospital of Beijing in the city-level data. We will seek the comparison between these hospitals in future studies. The 3 years of the post-implementation period seem just acceptable to assess the DIT, and studies of longer follow-up are much desired. Second, studies with more rigorous study design, such as case-control and cohort studies, are needed to validate the findings of this descriptive study. Third, we cannot exclude that the factors other than universal ZMDP also contributed to the effects of the universal ZMDP shown by this study. Fourth, the inpatient mortality without case severity as reported here may not be very informative and can sometimes be misleading. However, our survey revealed that some published works reported inpatient mortality with case severity,[13, 25, 26] and some without.[16, 27, 28] A systematic review indeed showed that only 1 of 15 (7%) studies used disease severity index in reporting inpatient mortality of interdisciplinary team care.[28] Nonetheless, cautions should be used when applying our findings on inpatient mortality changes. Finally, there were no individual-level data, which were not available due to governmental restrictions. These data, if available, would help validate our findings and provide more insights into the behavioral changes of the hospitals, physicians and patients after the implementation of the universal ZMDP.

In conclusion, our data demonstrate that the implementation of universal ZMDP in an urban tertiary hospital had sustainable impacts on its annual patient-visit volumes and RMOH, but different effects on the inflation-adjusted per-visit/discharge medicine charges of the outpatient and inpatient services. We also find the universal ZMDP may link to no significant changes in physician workload and inpatient mortality. Our findings may help with the future implementation and modification of the universal ZMDP.

## Supporting Information

S1 Table Comparison of the effects associated with the zero-markup policy implemented in Dec 2012 at the Jishuitan hospital (Student t test) Note: *Adjusted by the respective annual consumer product indices in China with that in 2009 as 100%; RMB, Reminbi; Physician workloads are average patient-visit number per day for outpatient service and average patient-bed number per day for inpatient service, respectively.
(DOC)
