# Supplementary material for: Descriptive Analysis on the Impacts of Universal Zero-Markup Drug Policy on a Chinese Urban Tertiary Hospital
Source: PLoS One. 2016 Sep 14;11(9):e0162795. doi: 10.1371/journal.pone.0162795 (PMC5023112; doi:10.1371/journal.pone.0162795)
Supplement: S1 Table — Note: *Adjusted by the respective annual consumer product indices in China with that in 2009 as 100%; RMB, Reminbi; Physician workloads are average patient-visit number per day for outpatient service and average patient-bed number per day for inpatient service, respectively. (DOC) [file pone.0162795.s001.doc]

S1 Table. Comparison of the effects associated with the zero-markup policy implemented in Dec 2012 at the Jishuitan hospital (Student *t* test)

|  | **2009-2012** |  | |  | **2013-2015** | |  |  |  |
| --- | --- | --- | --- | --- | --- | --- | --- | --- | --- |
| **Variable** | Mean | Std. Dev. | Min | Max | Mean | Std. Dev. | Min | Max | *P* value |
| ***Outpatient*** |  |  |  |  |  |  |  |  |  |
| **Volume** | 1122467 | 106759 | 1003473 | 1238676 | 1741767 | 191778.8 | 1527099 | 1896189 | 0.0027 |
| **Healthcare charge (RMB)*** | 372.875 | 18.00853 | 346.81 | 386.41 | 453.7467 | 40.878 | 407.29 | 484.21 | 0.0155 |
| **Medicine charge (RMB)*** | 239.15 | 11.93348 | 221.97 | 249.19 | 215.66 | 8.025004 | 209.19 | 224.64 | 0.0331 |
| **RMOH** | 0.64125 | 0.006994 | 0.633 | 0.65 | 0.477333 | 0.0321455 | 0.454 | 0.514 | 0.0002 |
| **Physician workload** | 6.975 | 0.222186 | 6.72 | 7.24 | 10.44667 | 0.6807591 | 9.67 | 10.94 | 0.0002 |
| ***Inpatient*** |  |  |  |  |  |  |  |  |  |
| **Volume** | 32094 | 5147.032 | 27327 | 38943 | 47692.67 | 4439.465 | 42622 | 50880 | 0.0086 |
| **Healthcare charge (RMB)*** | 27468.75 | 2425.781 | 24856.15 | 30683.54 | 27378.31 | 1587.304 | 26095.5 | 29153.44 | 0.9578 |
| **Medicine charge (RMB)*** | 6413.878 | 306.4277 | 6038.68 | 6789.26 | 4341.187 | 462.2497 | 3912.11 | 4830.67 | 0.0008 |
| **RMOH** | 0.2345 | 0.018303 | 0.219 | 0.258 | 0.159333 | 0.0255016 | 0.134 | 0.185 | 0.0059 |
| **Physician workload** | 2.3525 | 0.187506 | 2.1 | 2.5 | 2.536667 | 0.1365039 | 2.38 | 2.63 | 0.2129 |
| **Mortality** | 0.007275 | 0.00179 | 0.0049 | 0.0088 | 0.003433 | 0.000551 | 0.0028 | 0.0038 | 0.0169 |
| **Gross loss over total expenses** | -11.438% | 2.265% | -13.590% | -8.421% | -14.977% | 2.035% | -17.309% | -13.562% | 0.0865 |

Note: *Adjusted by the respective annual consumer product indices in China with that in 2009 as 100%; RMB, Reminbi; Physician workloads are average patient-visit number per day for outpatient service and average patient-bed number per day for inpatient service, respectively.
